# Fish consumption and its motives in households with versus without self-reported medical history of CVD: A consumer survey from five European countries

**DOI:** 10.1186/1471-2458-8-306

**Published:** 2008-09-10

**Authors:** Zuzanna Pieniak, Wim Verbeke, Federico Perez-Cueto, Karen Brunsø, Stefaan De Henauw

**Affiliations:** 1 Department of Agricultural Economics, Ghent University, Coupure links 653, 9000, Gent, Belgium; 2 MAPP Centre, University of Aarhus, Haslegaardsvej 10, DK-8210, Aarhus, Denmark; 3 Department of Public Health, Ghent University, De Pintelaan 185, 9000, Gent, Belgium

## Abstract

**Background:**

The purpose of this study was to explore the cross-cultural differences in the frequency of fish intake and in motivations for fish consumption between people from households with (CVD+) or without (CVD-) medical history of cardiovascular disease, using data obtained in five European countries.

**Methods:**

A cross-sectional consumer survey was carried out in November-December 2004 with representative household samples from Belgium, the Netherlands, Denmark, Poland and Spain. The sample consisted of 4,786 respondents, aged 18–84 and who were responsible for food purchasing and cooking in the household.

**Results:**

Individuals from households in the CVD+ group consumed fish more frequently in Belgium and in Denmark as compared to those in the CVD- group. The consumption of fatty fish, which is the main sources of omega-3 PUFA associated with prevention of cardiovascular diseases, was on the same level for the two CVD groups in the majority of the countries, except in Belgium where CVD+ subjects reported to eat fatty fish significantly more frequently than CVD- subjects. All respondents perceived fish as a very healthy and nutritious food product. Only Danish consumers reported a higher subjective and objective knowledge related to nutrition issues about fish. In the other countries, objective knowledge about fish was on a low level, similar for CVD+ as for CVD- subjects, despite a higher claimed use of medical information sources about fish among CVD+ subjects.

**Conclusion:**

Although a number of differences between CVD- and CVD+ subjects with respect to their frequency of fish intake are uncovered, the findings suggest that fish consumption traditions and habits – rather than a medical history of CVD – account for large differences between the countries, particularly in fatty fish consumption. This study exemplifies the need for nutrition education and more effective communication about fish, not only to the people facing chronic diseases, but also to the broader public. European consumers are convinced that eating fish is healthy, but particular emphasis should be made on communicating benefits especially from fatty fish consumption.

## Background

Fish and seafood products are recommended to take a prominent position in the human diet due to their beneficial role in the prevention of chronic degenerative diseases. The consumption of fish may be protective against certain cancers [1-4] and cardiovascular diseases [5,6]. Consumption of fish or fish oils lowers the risk of coronary heart disease, death or sudden death [7-10]. This (health) beneficial role of fish intake is particularly due to its omega-3 polyunsaturated fatty acids (PUFA) content, which have been associated with the prevention of cardiovascular diseases [10]. Eicosapentaenoic acid (EPA) and docosahexaenoic acid (DHA) that are formed from alpha-linolic acid have been identified as the two long-chain omega-3 PUFA's to be the likely active constituents of fatty fish. EPA has protective health effects such as the lowering rates of heart diseases [11], the reduction of arrhythmias and thrombosis [12], the lowering plasma triglyceride levels [13], and the reduction of blood clotting tendency [14,15].

Previous studies carried out on general population samples found that attitudes towards fish consumption [16,17], motivational aspects such as health involvement or the importance attached to healthy eating [18,19], were significant factors in explaining fish consumption. Also people's health motivation and knowledge about nutrition were, among others, positive predictors of dietary health preventive behaviour [20]. Furthermore, fish availability, perceived difficulty or easiness in the preparation and cooking of fish, perception that fish is expensive compared to the other food types, physical properties such as bones and smell, and taste preference were found to be important factors shaping fish consumption [21-25]. Despite the predominantly healthy image fish has among nutritionists, food scientists, government and consumers [26-28], the recommendations of eating fish at least twice a week are not met by large groups of the population in many countries [29,30].

## Scope and objectives

Our specific interest for performing this study stems from the role of fish consumption in the prevention of cardiovascular disease (CVD). The aim of this paper is to investigate the differences in the frequency of fish consumption and motivational aspects such as health beliefs, use of and trust in information sources and knowledge levels, between individuals from households with (CVD+) versus without (CVD-) medical history of CVD in Belgium, Denmark, The Netherlands, Poland and Spain.

The risk of CVD can be lowered by adhering to dietary and lifestyle recommendations [31], particularly the weekly consumption of two portions of fish, one of which should be fatty fish. Therefore, at least if dietary recommendations were consciously adhered to, one would expect that individuals from CVD+ households will report a higher frequency of fish consumption in general and fatty fish consumption in particular, as compared to those who have not been confronted with CVD in their direct social environment. As it is known, people are influenced by others, also in their food purchase and consumption behaviour. Consumers having the feeling that other people who are important to them, such as family members, stimulate their consumption are also reporting a higher intention to buy fish [17,19]. Additionally, the moral obligation or personal norms of individuals may lead to performing a particular behaviour for reasons other than own liking, like serving the family a healthy meal [19,21]. Therefore, we believe that questioning people responsible for the shopping in their household is relevant and meaningful.

Whether dietary recommendations are adhered to is likely to depend also on multiple other personal factors, including cultural background, as well as attitudinal and information-related variables. In this study, we will concentrate on countries with a weak (Poland), a moderate (Belgium, the Netherlands and Denmark) and a strong tradition (Spain) of eating fish. Based on the Food Balance Sheets data provided by FAO [32], Spain reported one of the highest fish intakes in Europe and in the world with 45 kg/capita/year, whereas Belgium, Denmark and the Netherlands reported moderate fish consumption levels of 24, 23 and 23 kg/capita/year, respectively, close to the EU average based on the 27 countries (21.4 kg/capita/year). Poland was among the countries with the lowest consumption of fish within Europe (9 kg/capita/year). Determinants of fish consumption might be different depending on the country and fish consumption level. What is important in one country may not be significant in the other. Therefore, some cross-cultural differences related to motivational aspect of fish consumption are expected.

The use of information sources as a part of information search [33,34] in the decision making process [35], associates with behaviour and/or food choice [36]. Therefore, information addressed to consumers must be reliable and trustworthy [37], since trust plays a crucial role in the utilisation of provided information [38] and is an important antecedent to information effectiveness. Furthermore, consumer knowledge has been proven to be a relevant and significant construct that influences how consumers gather and organise information, and ultimately, what products they buy and how they use them [39]. In this study, two knowledge constructs will be distinguished: subjective knowledge and objective knowledge [34,40-42]. Subjective knowledge relates to people's perceptions of what or how much they know about a product class and are based on consumer's interpretation of what s/he knows, while objective knowledge refers to the accurate information about the product class stored in long-term memory [42,43].

## Methods

### Study design

This study was part of the consumer science pillar of the Integrated Project SEAFOODplus (FOOD-CT-2004-506359) , which has been approved for funding under the EU Sixth Framework Programme. The overall research design for this study has been described in detail elsewhere [44] and will only be summarised here. Cross-sectional survey data were collected through questionnaires in five European countries: Belgium, Denmark, The Netherlands, Poland and Spain during November-December 2004. All relevant international guidelines and standards relating to the collection of personal data from human beings have been abided. Participants in the consumer studies were adult volunteers from whom written informed consent has been obtained. The data collection fieldwork has been performed by professional market research agencies who have agreed to abide the ICC/ESOMAR International Code on Market and Social Research [45]. This code embodies the highest professional and ethical standards relating to market and social research, and guarantees amongst other things, informed consent and the anonymous processing of personal data.

Sample selection and contact procedures differed between countries, depending on cost efficiency, time effectiveness and best practice of the market research agencies that performed the fieldwork. Households were selected at random, either from panels (Belgium and The Netherlands), phone books (Denmark), census data (Poland) or through random walk procedures (Spain), taking predetermined quota with respect to age and regional distribution into account within each country. In Denmark and Belgium, the field work consisted of mail surveying with a response rate of 79% and 53%, respectively. In Poland and in Spain, the participants were contacted face-to-face at their homes. Upon their agreement to participate, they were asked to self-administer and return the completed questionnaire. In The Netherlands, data were collected electronically by means of a web-based survey. Most importantly, all questionnaires were self-administered by the participants without interference from the researchers, the agency or interviewers. The dataset used contained only fully anonymous and non-identifiable records.

### Measures

A questionnaire was developed in English and further translated into Dutch and French (Belgium), Danish (Denmark), Dutch (The Netherlands), Polish (Poland) and Spanish (Spain) by professional translation service in each country. The back-translation method was used to construct the local language versions of the questionnaire. The questionnaires, measuring a wide variety of constructs including behaviour, attitudes and beliefs, knowledge, and use of information sources, have been pre-tested in the national languages through pilot studies.

In order to obtain a measure for whether an individual has been confronted with CVD in her/his direct social environment, respondents were asked if there was anybody in their households suffering or having suffered from cardiovascular diseases (nominal yes/no scale). In this study household refers to all individuals who live in the same dwelling. It has been translated into "gezin" (Flemish/Dutch), "foyer" (French), "gospodarstwo domowe" (Polish), "husstanden" (Danish), and "hogar" (Spanish). No concrete definition of cardiovascular diseases has been given to the respondents. We assumed that cardiovascular diseases refer to the class of diseases that involve the heart and/or blood vessels (arteries and veins). Nevertheless, we used the term that is the most familiar in common layman language in the respective countries. From a medical perspective these terms do not necessarily cover all potential diseases involving heart and/or blood vessels. From our perspective, it is the subjective feeling, or even reality of facing or having faced any disease related to cardiovascular diseases that matters. Our measure is a self-reported, single item measure that indicates if there are/were persons in the households with a medical history of cardiovascular diseases. It is important to note that no medical examinations have been carried out in this study. In order to avoid post-rationalisation and social desirability response behaviour, this question about CVD was asked at the end of the questionnaire.

Fish consumption behaviour was a self-reported measure and was scaled as the frequency of total fish consumption per week. The respondents were asked through two questions how often they eat fish both at home and out of home; then the responses were summated in order to create one final variable, namely, total fish consumption. Additionally, the consumption of fatty fish was assessed by measuring the consumption frequency of four fatty fish species (fat concentration > 10% on fresh weight basis): salmon, mackerel, eel and herring. A 9-point frequency scale ranging from "never" (1) to "daily or almost every day" (9) was used for both total and fatty fish consumption. Assuming that fish consumption behaviour might be driven through dietary recommendations related to the prevention against cardiovascular diseases, these variables were recoded into binary (yes/no) fish consumption variables; either meeting or not meeting dietary recommendations with respect to fish. In the case of total fish consumption, the benchmark (dietary recommendation) is eating fish at least twice a week; whereas for fatty fish it is fatty fish intake at least once a week.

Perception of fish as being a healthy food was measured by two items: "Eating fish is healthy", and "Eating fish is nutritious". Both statements were scored on a 7-point Likert scale, ranging from "totally disagree" (1) to "totally agree" (7).

Satisfaction with life was measured using the scale developed by Diener [46] and consisted of four items. Five items regarding subjective health were included. The items were mainly based on the general health perception scale from the short-form health survey SF-36 [47]. Interest in healthy eating was measured by five items adapted from the Food Choice Questionnaire [48]. Only the most appropriate, useful and relevant items related to fish were included based on findings from exploratory focus group discussions [33,49]. Health involvement consisted of three items based on the involvement scale developed by Zaichkowsky [50], which also corroborates the food involvement scale suggested by Bell and Marshall [51]. Those four constructs have been cross-culturally validated across the consumer samples taken from Belgium, The Netherlands, Spain, Denmark and Poland [52].

Next, respondents were asked about their use of different information sources in order to obtain information about fish. Only information sources communicating about public health issues were selected for the current analysis, such as doctor, dietician, public health recommendations, government and scientists. A 7-point Likert scale ranging from "never" (1) to "very often" (7) was used. Next, consumers' trust in those sources was assessed [53]. Respondents were asked to rate each of the above mentioned information sources to the question "To what extent do you trust information about fish from the following sources?" on 7-point Likert scales ranging from "completely distrust" (1) to "completely trust" (7).

Subjective knowledge about fish was measured by three items: (1) "My friends consider me as an expert on fish"; (2) "I have a lot of knowledge of how to prepare fish for dinner"; and (3) "I have a lot of knowledge how to evaluate the quality of fish" to be answered on a 7-point Likert scale ranging from "totally disagree" to "totally agree"; consistent with measures used in previous studies [e.g. [34,42]].

Next, consumer's level of objective knowledge about fish and cardiovascular diseases was measured with three statements that are either true or false. It was assumed that those statements should be common knowledge among at least half of the population. One of the statements was false: "Cod is a fatty fish" (cod is classified as a lean fish) and two statements were true: "Fish is a source of omega-3 fatty acids"; and "Salmon is a fatty fish". For the three statements, a binary scale "true"/"false" was used [42]. We opted for not including a "don't know" response category, which forced respondents to think and make up their mind about the proposed statements.

### Participants

A total sample of 4,786 consumers (n = 800–1,100 respondents per country) was obtained. The sample was composed of 3,652 women (76.3%) and 1,134 men (23.7%). This gender distribution reflects the criterion that all respondents were the main responsible people for food purchasing within their household. The age of the respondents ranged from 18 to 84 years, with a mean of 42.7 (SD = 12.6). The main socio-demographic characteristics of the participants from each of the five European countries are presented in Table 1.

**Table 1:** Sample characteristics (%)

	Belgium (n = 852)	Denmark (n = 1,110)	Netherlands (n = 809)	Poland (n = 1,015)	Spain (n = 1,000)	Total (n = 4,786)
*Gender*						
Male	24.8	25.6	28.4	30.0	10.6	23.7
Female	75.2	74.4	71.6	70.0	89.4	76.3
						
*Age*						
< 25 years	3.2	3.6	14.5	11.0	19.5	10.2
25–55 years	73.0	70.7	71.3	70.7	69.0	70.9
> 55 years	23.8	25.7	14.2	18.3	11.5	18.9
						
*Education*						
Unskilled	20.2	46.8	45.2	43.7	62.6	44.4
Skilled	71.2	38.2	49.9	43.4	11.5	41.6
Higher	8.6	15.0	4.9	12.9	25.9	14.0
						
*Household income*						
Lower	25.8	21.8	30.2	30.0	21.9	25.7
Middle	49.6	47.9	49.2	43.6	49.3	47.8
Upper	24.6	30.3	20.6	26.4	28.8	26.5

### Statistical analyses

Data were analysed using SPSS version 15. First, in order to validate the health related scales an exploratory factor analysis with the seventeen items related to health was performed. A maximum likelihood extraction method followed by a Promax rotation yielded four factors. The eigenvalues of the first five factors of the unrotated solution were 5.66, 3.42, 1.76, 1.30 and 0.87. We decided to extract four factors (71.4% of the total variance) as they gave the most parsimonious solution, accounting for 63.9% of the variance after Promax rotation. Table 2 presents the factor loadings, percentage of variance explained, the internal consistency reliability of the four resulting health constructs, as well as their mean and standard deviation for the total sample. The reliability coefficients alpha indicate that the different items with a high loading on a specific factor can be summated into a composite construct score. The four constructs are further referred to as 'Interest in healthy eating', 'Satisfaction with life', 'Health involvement' and 'Subjective health'.

**Table 2:** Validity of the health constructs (n = 4,786)

Health constructs and indicators	Factor 1	Factor 2	Factor 3	Factor 4
*Interest in healthy eating*				
It is important to me that the food I eat on a typical day is good for my psychical and mental health	0.943			
It is important to me that the food I eat on a typical day keeps me healthy	0.863			
It is important to me that the food I eat on a typical day is nutritious	0.839			
It is important to me that the food I eat on a typical day is a natural product	0.633			
It is important to me that the food I eat on a typical day has been produced without preservatives or additives	0.616			
				
*Satisfaction with life*				
I am satisfied with my life		0.868		
The general conditions of my life are excellent		0.848		
In most ways my life is close to my ideal		0.752		
If I could live my life over, I would change almost nothing		0.669		
				
*Health involvement*				
Health is very important to me			0.930	
I care a lot about health			0.922	
Health means a lot to me			0.918	
				
*Subjective health*				
Compared with people at my age, my health is excellent				0.971
Compared with people at my age, my current physical health is excellent				0.885
I am as healthy as anyone I know at my age				0.650
Compared with people at my age, my current mental health is excellent		0.219		0.412
I consider myself as very health conscious	0.228			0.201
Explained variance (%)	28.79	19.48	9.61	6.24
Cronbach's alpha	0.88	0.85	0.94	0.82
Mean (standard deviation)	5.81 (1.06)	4.85 (1.26)	6.18 (1.15)	4.94 (1.24)

Bivariate analyses through chi-square association tests were performed. The Fisher exact test was applied when cell counts were less than 5. Comparison of mean scores was performed through independent samples t-tests and analysis of (co)variance F-tests with Tukey's honestly significant difference (HSD) post hoc comparisons. Pearson's correlation was used to detect differences in demographic characteristics, consumer beliefs, perception and fish consumption frequency between respondents who have been confronted with cardiovascular diseases in their family and those who have not. Results are presented in table format expressed as percentages, mean scores and standard deviations, including test statistic p-values. A p-value less than 0.05 was considered as statistically significant.

## Results

### CVD based consumer groups

Respondents were divided into two groups: one group consisting of people who reported to face or have faced cardiovascular disease in their household (n = 351; 7.3%), further referred to as "CVD+" respondents and the other group of people from households who claimed no medical history of cardiovascular diseases (n = 4,435; 92.7%), called "CVD-" respondents.

Table 3 shows the socio-demographic characteristics of respondents from households with versus without medical history of cardiovascular diseases among the countries. The self-reported (subjective) prevalence of CVD ranges from around 4% in Denmark and Spain to more than 10% in Belgium and The Netherlands. These results, of which the external validity will be discussed later on, are subject to potential impact from the varying number of relatives and the corresponding age ranges in the households of the respondents, on which no specific data were collected. Despite the relatively low shares of CVD+ subjects within the samples, the number of participants is substantial enough for performing statistical analyses.

**Table 3:** Sample characteristics for CVD+ and CVD- households in five European countries; % of respondents within each CVD group

	Belgium			Denmark			The Netherlands			Poland			Spain		
	
	CVD+ (n = 90) 10.5%	CVD- (n = 762) 89.5%	p	CVD+ (n = 45) 4.0%	CVD- (n = 1065) 96.0%	p	CVD+ (n = 88) 10.9%	CVD- (n = 721) 89.1%	p	CVD+ (n = 87) 8.6%	CVD- (n = 928) 91.4%	p	CVD+ (n = 41) 4.1%	CVD- (n = 959) 95.9%	p
Age *	52.1	45.1	< .001	56.3	45.4	< .001	46.8	39.4	< .001	50.8	42.0	< .001	40.1	38.2	.347
															
*Gender*			.172			.381			.319			.989			.735
Male	18.9	25.5		20.0	25.8		33.0	27.9		29.9	30.0		12.2	10.5	
Female	81.1	74.5		80.0	74.2		67.0	72.1		70.1	70.0		87.8	89.5	
															
*Education*			.405			.494			.106			.037			.258
Unskilled	25.5	19.6		42.9	47.0		55.2	44.1		56.3	42.5		56.1	62.9	
Skilled	66.7	71.7		35.7	38.3		42.5	50.8		35.6	44.2		19.5	11.2	
Higher	7.8	8.7		21.4	14.8		2.3	5.2		8.0	13.3		24.4	24.9	
															
*Household income*			.521			.527			. 248			. 183			.694
Lower	30.0	25.2		15.6	22.1		34.1	29.7		31.0	29.8		26.8	21.7	
Middle	44.4	50.3		48.9	47.9		40.9	50.2		50.6	43.0		43.9	49.5	
Upper	25.6	24.5		35.5	30.0		25.0	20.1		18.4	27.2		29.3	28.8	

* Mean (years)

With regard to age, in Belgium, Denmark, The Netherlands and Poland CVD+ subjects were significantly older than the other group of respondents. No significant differences with respect to gender and income were found between the two groups. In Poland, the group of CVD+ consisted of significantly more individuals with lower education level (unskilled) and less higher educated and skilled individuals compared with the CVD- group. A similar tendency was observed for the Dutch respondents (p = 0.106) although this association was not statistically significant.

### Fish consumption frequency

Table 4 presents a comparison of the frequency of fish intake among CVD+ and CVD- households within and between the countries. In general, Spanish CVD+ and CVD- respondents reported the highest total fish consumption frequency, followed by Danes and Poles. Belgians and Dutchmen scored significantly lower than consumers from any other country. Polish and Danish respondents from both groups of CVD displayed the highest, whilst Belgians the lowest fatty fish consumption frequencies.

**Table 4:** Frequency of fish intake among CVD+ and CVD- households; comparison within and between the countries (n = 4,786)

	Belgium			Denmark			The Netherlands			Poland			Spain		
	
	CVD+ (n = 90) 10.5%	CVD- (n = 762) 89.5%	p	CVD+ (n = 45) 4.0%	CVD- (n = 1065) 96.0%	p	CVD+ (n = 88) 10.9%	CVD- (n = 721) 89.1%	p	CVD+ (n = 87) 8.6%	CVD- (n = 928) 91.4%	p	CVD+ (n = 41) 4.1%	CVD- (n = 959) 95.9%	p
Total fish (≥ 1/week)	67.8	50.1	.002	80.0	47.6	< .001	37.5	37.7	.967	54.0	54.8	.882	92.7	88.6	.421
Total fish (≥ 2/week)	33.3	20.7	.006	46.7	24.0	.001	26.1	16.8	.030	24.1	22.7	.766	82.9	71.1	.101
Fatty fish (≥ 1/week)	23.3	13.0	.008	42.2	32.6	.178	22.7	20.9	.699	47.1	41.9	.347	24.4	27.0	.711

The numbers indicate the percentage of respondents within each CVD group who eat fish in total at least once a week; at least twice a week; and fatty fish at least once a week. Mean values of fish intake were significantly different between countries for both CVD+ and CVD- (P < 0.001).

In Belgium, CVD+ respondents reported a significantly higher frequency of fish intake in general, and a higher frequency of fatty fish intake in particular. Almost 70% of CVD+ respondents claimed to eat fish at least once a week versus only half of CVD- respondents. One third of the CVD+ respondents versus one fifth of CVD- respondents reported fish intake in accordance with dietary recommendations, i.e. at least twice per week. With regard to fatty fish consumption, almost one quarter of the CVD+ respondents claimed to eat salmon, herring, mackerel and/or eel at least once a week, versus only 13% of CVD- respondents.

In Denmark, significant differences in the frequency of total fish consumption were found between the two CVD groups. About 80% of respondents from households with medical history of CVD indicated to consume fish at least once a week versus less than half of the CVD- respondents. Furthermore, almost half of the CVD+ respondents met the dietary recommendations of eating fish twice a week versus only one quarter of the CVD- respondents. No significant difference between CVD+ and CVD- with respect to fatty fish consumption was observed.

In The Netherlands, significantly more consumers from CVD+ households reported fish consumption at least twice a week in comparison with CVD- respondents. However, no significant differences in meeting the weekly fish consumption level were found between the two CVD groups. Comparing with the other countries, the smallest proportion of compliers with dietary recommendations was found in The Netherlands.

In Poland, no significant differences in the frequencies of fish consumption were found between CVD+ and CVD- respondents.

Finally, in Spain, no significant differences in the frequencies of fish consumption between the two groups were found. However, opposite to the Polish respondents, Spanish consumers reported a very high total fish consumption level, with about 90% of the respondents claiming to eat fish at least once a week. Furthermore, 82.7% of the CVD+ versus 71.1% of the CVD- consumers stated to consume fish minimum twice a week, thus meeting the dietary recommendations with respect to fish consumption.

### Potential factors influencing the frequency of fish intake

Table 5 presents the results of the analysis of covariance (ANCOVA) performed for potential factors influencing the frequency of fish intake between CVD+ and CVD- respondents within each of the five countries and across the countries. Analysis of covariance was used to compare potential factors influencing the frequency of fish consumption by state controlling for age (in all countries) and education (in the case of Poland). Hence, this analysis yields the effects after removing the variance for which the covariates age and education account.

**Table 5:** Mean values of potential factors influencing frequency of fish intake between CVD+ and CVD- households; comparison within and between the countries (n = 4,786)

	Belgium #				Denmark †				The Netherlands ‡			
	
	CVD+ (n = 90)		CVD-(n = 762)		CVD+ (n = 45)		CVD- (n = 1065)		CVD+ (n = 88)		CVD- (n = 721)	
	
	Mean	SD	Mean	SD	Mean	SD	Mean	SD	Mean	SD	Mean	SD
Eating fish is healthy	6.25	1.21	6.09	1.21	6.61	0.65	6.37	1.00	5.98	1.57	5.99	1.33
Eating fish is nutritious	5.70	1.54	5.74	1.34	6.44	0.84	6.29	1.04	5.78	1.42	5.60	1.31
												
Satisfaction with life	4.47	1.50	4.78	1.29	5.05	1.13	5.36	1.17	4.91	1.24	4.82	1.19
Subjective health	4.32	1.34	4.69	1.15	5.13	1.41	5.28	1.26	4.59	1.52	4.76	1.29
Interest in healthy eating	6.12	0.90	5.78	0.99	6.18	0.81	5.54	1.09	5.67	1.13	5.26	1.10
Health involvement	6.50	0.94	6.35	1.14	6.29	0.96	5.88	1.12	6.03	1.64	6.10	1.29
												
*Information sources*												
Use of medical	2.99	1.64	2.48	1.43	2.77	1.44	2.07	1.18	3.07	1.65	2.08	1.28
Use of non-medical	2.21	1.37	1.99	1.27	1.93	1.31	1.79	1.19	2.16	1.45	1.91	1.21
Trust in medical	5.00	1.22	4.84	1.41	5.14	1.44	5.00	1.24	4.62	1.31	4.68	1.25
Trust in non-medical	4.19	1.44	4.26	1.43	4.29	1.48	4.61	1.27	4.10	1.24	4.30	1.27
												
Objective knowledge	2.47	1.12	2.42	1.14	3.40	0.78	3.09	1.01	2.27	1.01	2.18	0.99
Subjective knowledge	3.72	1.66	3.13	1.48	4.04	1.77	3.38	1.64	2.94	1.52	2.91	1.55

	Poland§				Spain¶							
					
	CVD+ (n = 87)		CVD- (n = 928)		CVD+ (n = 41)		CVD-(n = 959)					
					
	Mean	SD	Mean	SD	Mean	SD	Mean	SD				

Eating fish is healthy	6.42	1.18	6.45	0.99	6.51	1.12	6.24	1.07				
Eating fish is nutritious	6.33	1.06	6.16	1.15	6.10	1.50	6.22	1.03				
												
Life satisfaction	4.49	1.26	4.38	1.25	4.31	1.25	4.89	1.15				
Subjective health	4.60	1.41	4.77	1.26	5.06	1.18	5.16	1.04				
Interest in healthy eating	6.35	0.87	6.14	0.95	6.21	0.68	6.12	0.91				
Health involvement	6.33	1.20	6.28	1.15	6.43	0.89	6.29	0.99				
												
*Information sources*												
Use of medical	3.62	1.72	3.04	1.77	4.00	1.67	3.58	1.64				
Use of non-medical	1.70	1.03	1.73	1.11	2.34	1.50	2.23	1.44				
Trust in medical	4.52	1.76	4.05	1.84	5.46	1.15	5.17	1.24				
Trust in non-medical	2.38	1.37	2.48	1.38	4.30	1.43	4.03	1.40				
												
Objective knowledge	2.21	1.08	2.17	1.03	2.71	0.95	2.57	0.94				
Subjective knowledge	3.83	1.45	3.71	1.48	3.98	1.29	3.83	1.37				

Mean values of all constructs were significantly different between countries for CVD+ (P < 0.001) after adjusting for age (in all countries) and education (only in Poland) (ANCOVA). Mean values were significantly different between countries for CVD- for all constructs (P < 0.001), except for subjective health, health involvement and use of independent information sources (ANCOVA).

# Significant differences between CVD groups in Belgium for life satisfaction (P = 0.018), subjective health (P < 0.001), use of medical info sources (P < 0.05) and subjective knowledge (P = 0.021) (ANOVA).

† Significant differences between CVD groups in Denmark for subjective health (P = 0.014) and use of medical info sources (P = 0.002) (ANOVA).

‡ Significant differences between CVD groups in the Netherlands for healthy eating (P = 0.037) and use of medical info sources (P < 0.001) (ANOVA).

§Significant differences between CVD groups in Poland for use of medical info sources (P = 0.014) and trust in medical info sources (P = 0.039) (ANOVA).

¶Significant differences between CVD groups in Spain for life satisfaction (P = 0.001) (ANOVA).

Both groups of respondents scored very high on the health beliefs related to fish consumption, meaning that all respondents perceived fish as a very healthy and nutritious food product. In general, no significant differences in the belief that eating fish is healthy and nutritious between the two consumers groups were found for the majority of the countries. Comparison of the beliefs across the countries showed that in both groups of CVD respondents, Polish, Danish and Spanish respondents were most positive, whereas Belgian and Dutch respondents were least persuaded of the healthy and nutritious properties of fish.

Three personal self-reported health constructs were included in the analysis, namely satisfaction with life, subjective health and health involvement. In Belgium and Spain, CVD- respondents were significantly more satisfied with their life compared to the CVD+ respondents. In Denmark the difference in life satisfaction between the two groups was marginally significant (P = 0.056), with CVD+ respondents scoring higher. Spanish CVD+ respondents were the least satisfied with their life, whereas Danish and Dutch CVD+ consumers were the most satisfied with life. With regard to people who did not report to face or have faced cardiovascular disease in their household, Danish and Spanish respondents scored highest, whilst Belgians and Polish lowest on life satisfaction.

In Belgium and Denmark, CVD- respondents scored significantly higher on subjective health than CVD+ respondents, meaning that the first perceive themselves as much healthier. Subjective health perception was not significantly different in the Netherlands, Poland and Spain between the two groups. In both CVD groups Danish and Spanish consumers considered themselves healthier as compared to people from Belgium, The Netherlands and Poland. Further analyses were undertaken to investigate whether the health constructs were correlated with each other. The present study found strong correlations (p < 0.001) between satisfaction with life and subjective health (r = 0.511 in Belgium; r = 0.515 in Denmark; r = 0.581 in The Netherlands; r = 0.500 in Poland; and r = 0.439 in Spain).

Interest in healthy eating was significantly different between the two groups in The Netherlands and marginally significant in Denmark (P = 0.056). In those countries, consumers facing the CVD in their direct social environment were substantially more interested in healthy eating. Although in general, the respondents from all countries were interested in healthy eating, some differences were noticed. CVD+ and CVD- consumers from Poland and Spain attached most interest to healthy eating, whereas consumers from The Netherlands displayed the lowest interest in healthy eating.

Finally, no significant difference between the two groups of respondents on health involvement was detected. Remarkably, all respondents scored very high on the health involvement construct, meaning that their personal health is evaluated as very important to them. Consumers from both CVD groups from Belgium, Spain and Poland were the most involved with health, whereas Danish and Dutch consumers showed the lowest involvement with health.

In Belgium, Denmark, The Netherlands and Poland, CVD+ respondents indicated to use significantly more medical information sources about fish, such as doctor, dietician, and public health recommendations than CVD- respondents. Spanish and Polish respondents from both groups of CVD households reported the highest use of medical information sources, whereas Danish the lowest. The use of non-medical information sources, such as government and scientists, was not significantly different between CVD+ and CVD- in any of the countries but significantly different across the countries. Spaniards and Belgians used non-medical information sources the most frequent, while Poles and Danes least often. Remarkably, the scores on the use of medical information sources were much higher than the scores on the use of non-medical information sources. Doctor, dietician and public health recommendations were more frequently used as sources of information than government and scientists. With respect to trust in information sources, a significant difference between the two CVD groups was found only in Poland. CVD+ respondents reported significantly higher trust in medical information sources than individuals without medical history of cardiovascular diseases. However in general, Polish respondents held the lowest trust in all information sources compared with the other countries.

A marginally significant (P = 0.085) difference in the objective, thus factual knowledge related to fish (with relevance to its nutritional nature) was found in Denmark between the two consumer groups. CVD+ respondents reported substantially higher objective knowledge about these fish aspects compared to CVD- subjects. Generally, Danish respondents (both CVD+ and CVD-) reported the highest, whereas Polish and Dutch respondents displayed the lowest objective knowledge. Only in Belgium, CVD+ respondents perceived themselves as more knowledgeable about fish than CVD- respondents. Danish CVD+ respondents and Spanish CVD- respondents evaluated themselves as with highest subjective knowledge about fish. Dutchmen and Belgians from both CVD groups perceived themselves as least knowledgeable.

Summing up, Belgian respondents from CVD+ households consumed fish significantly more frequently in general and fatty fish in particular. They reported more frequent use of medical sources of information. Additionally, they were more confident about their knowledge about fish, felt less healthy and less satisfied with their lives than CVD- respondents.

In Denmark, CVD+ respondents consumed fish significantly more frequently in general as compared to CVD- subjects. They were more interested in healthy eating and used more frequently doctor, dietician or/and public health recommendations as information sources about fish. Furthermore, they reported higher objective knowledge about fish, but they felt less healthy and less satisfied with their lives in comparison with the CVD- respondents.

In The Netherlands, both groups of respondents denoted very low fish consumption frequency levels. CVD+ consumers were more interested in healthy eating and reported higher use of medical information sources about fish than the other group of consumers.

Similarly to the Netherlands, Polish respondents reported a very low frequency of fish intake in general, but the highest frequency of fatty fish intake at least once a week. Individuals from households with a medical history of CVD in their households reported higher use of and trust in medical information sources.

Finally, Spanish respondents reported a very high fish consumption frequency. The only significant difference was found for the satisfaction with life construct. People from CVD- households reported to be more satisfied with their life in comparison with the CVD+ group.

## Discussion

### Fish consumption frequency

The purpose of this study was to explore the cross-cultural differences in the frequency of fish intake and motivation for fish consumption among people from households with versus without medical history of cardiovascular diseases, based on data from five European countries. The results confirm our expectation that more of the consumers from households with a medical history of cardiovascular diseases followed dietary recommendations related to the frequency of total fish intake, i.e. ate fish at least twice a week, in comparison with consumers without medical history of CVD. Nevertheless, only in Belgium and in Denmark the differences in the total fish intake between the two groups were significant. This study emphasises an enormous discrepancy in the frequency of fish consumption between Northern and Southern European countries. In Spain, almost three quarters of sample reported eating fish at least twice a week, whilst in the other countries only about one quarter did (Belgium 22.1%; Denmark 25.0%; The Netherlands 17.1%; and Poland 22.9%). With regard to frequency of fatty fish consumption, in most of the countries (except in Spain) more people with a medical history of CVD ate fatty fish at least once a week in comparison with people without medical history of CVD. Nevertheless, only in Belgium the difference was significant. Interestingly, the Polish sample includes the highest number of respondents who reported consuming fatty fish at least once a week compared to the respondents from the other countries. The explanation is that herring, which is a fatty fish, is a traditional fish consumed in Poland (mostly marinated), and its high consumption among both CVD+ and CVD- respondents resulted in a relatively high frequency of fatty fish consumption (more than 40% ate fatty fish at least once a week). Nevertheless, this higher fatty fish consumption level as compared to other countries does not translate into higher total fish intake. Although Spaniards display the highest fish consumption frequency, only about one quarter of Spanish respondents declared to eat fatty fish at least once a week. This implies that differences in the fatty fish consumption levels might not be the result of adherence to dietary recommendations, but rather reflecting a tradition of eating (predominantly lean) fish as a part of the Mediterranean diet in Spain [54] and high herring consumption level in Poland [55]. Although strong scientific evidence exists that lower risk of death due to coronary heart diseases is more strongly related to the intake of fatty fish rather than lean fish [56-59], consumers may not be aware of it. Only in Belgium significantly more CVD+ respondents consumed fatty fish at least once a week compared with CVD- respondents. These findings may indicate that for most of the respondents, fatty fish is not perceived as having particular health beneficial effects as compared to lean fish. Instead, the findings suggest that 'fatty fish' and 'fatty acids' might be rather associated with "fatty", thus high in fat, and therefore also less healthy or unhealthy. In a series of studies convincing evidence has been found [60-63] that foods acquire reputations of being good or bad; these reputations as well as "foods healthfulness" are greatly influenced by real or perceived fat content (food high in fat is believed to be unhealthy). Furthermore, a previous fish consumer study based on a Belgian consumer sample found that Belgian consumers held strong beliefs that regular fish consumption reduces risks for coronary heart disease, which is one of the cardiovascular diseases [29]. This could explain the difference observed in the frequency of fatty fish intake between both CVD groups in our Belgian sample.

### Motivational aspects for fish consumption among CVD+ versus CVD- subjects

In general, the results display significant differences between the countries in most of the investigated motivational aspects for fish consumption (except for the belief that eating fish is healthy). No significant differences in the beliefs about fish health and nutrition were found between the two groups in the majority of the countries. Both CVD+ and CVD- respondents perceive fish as a very healthy and nutritious food. This confirms previous reports demonstrating that fish has a healthy image among consumers [26,27,29,33,49]. Only in Denmark, consumers from CVD+ households perceived fish even healthier and more nutritious than the CVD- subjects.

In general, respondents from households with a medical history of cardiovascular disease, feel personally less healthy than the respondents not confronted with CVD in their household, as demonstrated by the CVD+ subjects' lower score on the subjective health construct. Nevertheless, only in Belgium this difference was significant. Our results confirm previous findings where subjective health was found to be routinely better among people with fewer illnesses [64-66]. Furthermore, Belgian (p = 0.068) and Danish (p = 0.086) and Spanish (p = 0.002) CVD+ respondents (tend to) feel less satisfied with their life than CVD- respondents. These findings are in agreement with previous reports where self-rated general health was found to associate with future health and people's satisfaction with life [67].

Recommendations about healthy eating have been shown to influence consumers' food-related beliefs and consumption patterns [68,69]. Perceptions and beliefs are shaped by knowledge, which in turn is a product of exposure to information sources and personal effort in obtaining information [70]. In this study, respondents from households with a medical history of CVD in almost all countries (except in Spain) reported substantially higher use of medical information sources. In Denmark, CVD+ respondents not only reported higher frequency of total and fatty fish consumption but also held stronger beliefs about fish health. Additionally, they displayed higher subjective and objective knowledge about fish in the context of cardiovascular diseases. However, this was the case only for Danish consumers. In the other countries, despite CVD+ subjects' higher claimed use of medical information sources about fish, their objective knowledge was on a low level, similar as for respondents from households without medical history of cardiovascular diseases. Research carried out on the general population in Poland found that men with coronary heart diseases and women with a family history of CVD death reported significantly higher levels of knowledge related to CVD prevention methods [71]. In our study, both groups of Polish respondents reported a very low level of objective knowledge about fish. With regard to Belgian consumers with medical history of CVD, despite a significantly higher subjective knowledge level, their objective, i.e. factual knowledge is on the same level as for CVD- respondents.

Previous research indicated that consumers face difficulties in understanding concrete information about dietary fat and that the majority of consumers is not particularly interested in knowing more on the subject [72,73]. This may explain, first, the low level of objective knowledge about fish in the context of cardiovascular diseases in the majority of the countries, and second, the fact that fatty fish and fatty acids are not consistently understood as beneficial for health. The latter may also result from awareness of the potential presence of, and toxicological risks posed by particular fat-soluble environmental contaminants such as PCBs and dioxins in fatty fish species. However, empirical findings indicate that consumers are either hardly aware of these risks, or they are not particularly concerned about contaminants in fish [52].

Some limitations of this study should be acknowledged. The most important limitation relates to the fact that we used a rather blunt measure for CVD prevalence. Respondents were grouped into those with versus without a medical history of cardiovascular diseases based on a single item measure. As a result, the data obtained on CVD prevalence could potentially suffer from some weakness in the external validity. The single question probing for CVD prevalence could have been interpreted in different ways in different countries and – within countries – also variations in the understanding and the conceptualisation of this category of diseases could have been occurred. A similar effect modification could have been introduced because of differences in the understanding and interpretation of these terms and concepts between social classes. These limitations might partially explain why the gradients in CVD prevalence do not perfectly match the picture known from longitudinal epidemiological studies that have measured CVD prevalence in a standardised way, like the WHO MONICA study [74,75]. From these studies, it is known that CVD prevalence in general increases from Southern Europe to the North and from Western Europe to the East. Nevertheless, the absence of any significant differences between sexes in the reported CVD prevalence suggests a similar understanding of the construct of the question on CVD; in the opposite case, a much higher prevalence would be expected in males. Overall, it can be concluded that the characterisation of individuals as a function of a medical history for CVD has not reached the highest accuracy level for this study, but at the same time there is an indication for sufficient discriminatory value in order to allow some carefully formulated conclusions in terms of knowledge, behaviour and attitudes related to fish consumption. Furthermore, this study focused only on health-related factors that are only one kind of driver of food choice, dietary habits and eating behaviour, such as fish consumption, and mostly not even the main one. Other factors, like taste, availability, convenience and price perceptions may account for substantial differences in fish consumption behaviour, and therefore, future research investigating the impact of such perceptions together with those investigated in the study is highly recommended.

Furthermore, the present study may face some limitations induced by the use of different sample selection, recruitment and contact procedures that were used across the countries. The choice of procedures was informed by best practice within each country. Although the difference in procedures may have introduced some bias, most important is that all questionnaires were self-administered by the participants, and that the procedures yielded samples that are representative for age and region within each country. Although some differences in the composition of the sample exist, more specifically with respect to the age distribution, the applied statistical analyses (ANCOVA) have allowed accounting for the variance induced by such covariates. Finally, it should be noted that the data were collected within a broader consumer survey, focusing mainly on motives, barriers and attitudes with respect to eating fish in Europe, and not on measuring actual fish intake. Inference was often drawn based on claimed and self-reported behaviour. These answers may be subjected to social desirability, post-rationalisation, and cognitive dissonance or consonance and hence may deviate from actual behaviour. Therefore, it is recommended to take the issues relating to attitudes and knowledge as covered in our study on board in future epidemiological studies.

## Conclusion

In Belgium and in Denmark, people from households with a medical history of cardiovascular diseases consumed fish more frequently as compared to people who were not confronted with CVD. Surprisingly, the consumption of fatty fish, which is the main source of omega-3 PUFA associated with the prevention of cardiovascular diseases [10], was on the same level for the two groups in the majority of the countries (except in Belgium). Despite higher use of medical information sources about fish and higher interest in healthy eating in most of the countries among CVD+ respondents, their objective or factual knowledge about fish was on the same level as the respondents of CVD- households. This might be the most likely reason why fatty fish consumption was not more elevated in this group as compared to the consumers from households without medical history of CVD. Clearly, fish consumption traditions and habits – rather than a medical history of CVD – account for large differences between the countries, particularly in fatty fish consumption, which is very obvious in the cases of Spain (rather lean fish consumption) and Poland (rather fatty fish consumption).

Only in Belgium, the CVD+ consumers reported a significantly higher frequency of total and fatty fish intake which could be mainly due to their highest health involvement, their higher subjective knowledge and more frequent use of medical sources of information. With regard to Danish CVD+ respondents, their higher frequency of total fish consumption might be due to their highest level of subjective and objective knowledge and their higher health involvement. In Poland, higher use of medical information sources about fish and higher interest in healthy eating did not result in a higher frequency of fish consumption. In Spain, the fish consumption frequency is on a very high level, independently of their motivational aspects. Clearly, eating (mainly lean) fish is strongly habitual and a part of the traditional Mediterranean diet in Spain.

This study exemplifies the need for nutrition education and more effective communication about fish, not only to the people facing chronic diseases, but also to the broader public. Consumers are convinced that eating fish is healthy, but on one hand, particular emphasis should be on communicating benefits from fatty fish consumption, as the results suggest that people might perceive "fatty" in general as negative. On the other hand, by communicating benefits from fatty fish consumption respondents may perceive this as receiving contradictory information (in the case of meat, "fatty" is associated with unhealthy), which has been shown to have a negative influence. Communicating effectively requires that the target population is identified and their specificities are well understood and taken into account so as to make information meaningful, useful and efficient. Therefore, further research to explore consumers' knowledge about fish is recommended. More specifically, research on subjects with a medical history of cardiovascular diseases with regard to their health perception of fish, relation between knowledge about content and role of omega-3 fatty acids in fish and prevention of cardiovascular diseases is needed in order to issue appropriate dietary recommendations and public health information for both CVD+ and CVD- subjects. Even so, further research is needed dealing with the impact of information on consumer decision-making in the specific case of fish consumption.

## Competing interests

The authors declare that they have no competing interests.

## Authors' contributions

ZP participated in the study design, performed the statistical analysis and drafted the manuscript. WV participated in the study design, contributed to the statistical analysis, helped drafting the paper and coordinated the project. FPC revised the manuscript for important intellectual content, participated in the interpretation of the results, and contributed to editorial review. KB participated in the study design, coordinated the project and revised the manuscript for important intellectual content. SDH helped in the interpretation of the data and revised the manuscript for its content. All authors read and approved the final manuscript.

## Pre-publication history

The pre-publication history for this paper can be accessed here:


